# The NISTmAb Reference Material 8671 lifecycle management and quality plan

**DOI:** 10.1007/s00216-017-0844-2

**Published:** 2018-02-12

**Authors:** John E. Schiel, Abigail Turner

**Affiliations:** 1 National Institute of Standards and Technology, Institute for Bioscience and Biotechnology Research, 9600 Gudelsky Dr, Rockville, MD 20850 USA; 2 Present Address: Medimmune, LLC, 55 Watkins Mill Rd, Gaithersburg, MD 20878 USA

**Keywords:** Reference material, NISTmAb, Monoclonal antibody, Biotherapeutic, Biopharmaceutical, System suitability, Biosimilar

## Abstract

Comprehensive analysis of monoclonal antibody therapeutics involves an ever expanding cadre of technologies. Lifecycle-appropriate application of current and emerging techniques requires rigorous testing followed by discussion between industry and regulators in a pre-competitive space, an effort that may be facilitated by a widely available test metric. Biopharmaceutical quality materials, however, are often difficult to access and/or are protected by intellectual property rights. The NISTmAb, humanized IgG1κ Reference Material 8671 (RM 8671), has been established with the intent of filling that void. The NISTmAb embodies the quality and characteristics of a typical biopharmaceutical product, is widely available to the biopharmaceutical community, and is an open innovation tool for development and dissemination of results. The NISTmAb lifecyle management plan described herein provides a hierarchical strategy for maintenance of quality over time through rigorous method qualification detailed in additional submissions in the current publication series. The NISTmAb RM 8671 is a representative monoclonal antibody material and provides a means to continually evaluate current best practices, promote innovative approaches, and inform regulatory paradigms as technology advances.

**Electronic supplementary material:**

The online version of this article (10.1007/s00216-017-0844-2) contains supplementary material, which is available to authorized users.

**Graphical abstract:**
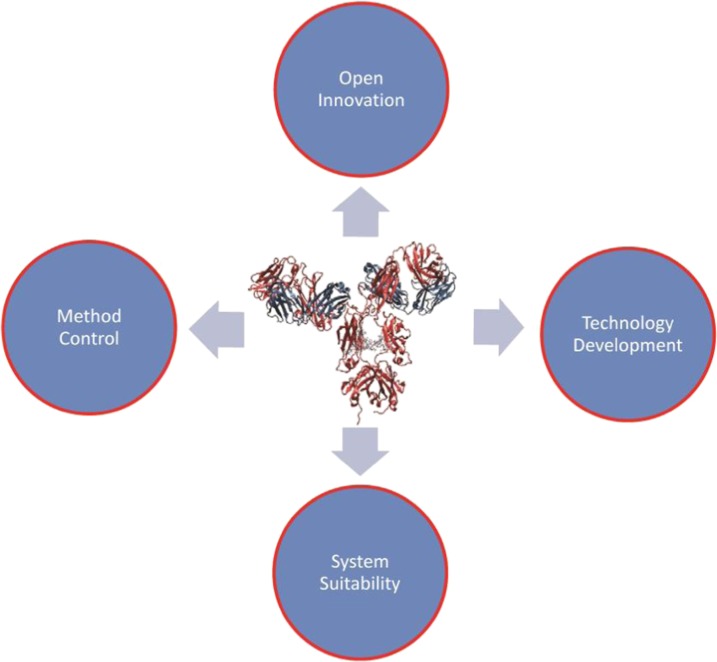
The NISTmAb Reference Material (RM) 8671 is intended to be an industry standard monoclonal antibody for pre-competitive harmonization of best practices and designing next generation characterization technologies for identity, quality, and stability testing.

## Introduction

Monoclonal antibodies increasingly dominate the biopharmaceutical market, boasting an unparalleled (and growing) diversity of indications such as cancer, hyperlipidemia, and autoimmune disorders [1]. In 2013, nearly 10 metric tons of monoclonal antibody were manufactured to supply nearly $75 billion in global sales [2]. Under the Quality by Design (QbD) paradigm, comprehensive physicochemical and biophysical characterization of product quality attributes (PQAs) provides the foundation for product development and eventual regulatory approval [3]. Notable technological advances driven by the need for deeper product understanding include the use of higher-order structural techniques (nuclear magnetic resonance [4–7], hydrogen-deuterium exchange (HDX) [7], and ion mobility mass spectrometry [8]), information rich application of multi-attribute mass spectrometry [9], high resolution glycoanalytical strategies [10], and novel technologies to identify and understand aggregation (transmission electron microscopy, x-ray scattering, and neutron scattering [11]). Imagine the transformative implications if such advanced characterization were amenable to broad scale screening of developability, real-time process feedback, or as an information rich and high-throughput release test. Consider the possibilities that would be afforded by a “fingerprint-like” biosimilarity assessment wherein complex dataset integration is made possible through fundamental understanding of method performance and product quality correlations [12]. The disconnect between “where we are” and “where we need to go” requires a pre-competitive test material of pharmaceutical-grade quality to comprehensively compare results between labs, evaluate analytical figures of merit, identify lifecycle-appropriate implementation points, reduce implementation risk, and facilitate regulatory assimilation [13].

The NIST monoclonal antibody (NISTmAb) is one such material, an IgG1κ that has been comprehensively characterized and intended as a platform for open innovation, collaboration, and development/evaluation of next generation technologies [13]. It offers an alternative to drug product-specific materials and data, which are not ideally suited for industry-wide harmonization and technology advancement because they are limited by intellectual property (IP) concerns, have limited accessibility and lot history, and have not been evaluated for suitability as a Reference Material (RM). The NISTmAb plays a unique, yet complimentary role compared to other publicly available standards [13]. The NISTmAb Reference Material 8671 (RM 8671) embodies the intrinsic structural features and heterogeneity of a drug substance produced using state-of-the-art bioprocess manufacturing, as well as the requisite quality characteristics to serve as an industry standard. The current publication provides context for the development of NISTmAb RM 8671 and describes the unique features of the NISTmAb characterization and lifecycle management program. The remaining series of publications in this set will further detail the extensive testing performed for the NISTmAb quality plan that will ensure its suitability as an industry standard [14–17].

### NISTmAb development history

The NIST, founded in 1901 as the National Bureau of Standards (NBS), is the national metrology institute of the United States. The original founding functions of the NBS were to include the custody of standards, described as “the construction when necessary of standards[…]the determination of physical constants, and the properties of materials when such data are of great importance to the scientific or manufacturing interest…” in a letter to Congress from Secretary of the Treasury Lyman J. Gage [18]. NIST continues its dedication to the development and use of standards as reflected in the current mission statement to promote innovation and industrial competitiveness by advancing measurement science, standards, and technology. A **Reference Material (RM)** is one such realization in the form of a material that is sufficiently homogeneous and stable with respect to specified properties [19, 20]. An RM must be established to be fit for its intended use in measurement or in examination of nominal properties. An RM may have property values reported; however, all known sources of uncertainty may not be fully accounted for. A **Certified Reference Material (CRM)**, in extension, is an RM providing one or more specified property values with all known associated uncertainty evaluated and a statement pertaining to metrological traceability [19, 20] (Standard Reference Materials, SRMs, are CRMs produced by NIST). RMs and CRMs must meet the ISO definitions for homogeneity, stability, and suitability for thier intended measurement process [20–22].

Every NIST RM and SRM is developed according to the NIST Quality Management system, which is based upon ISO 17034 [21, 23]. The current catalog of NIST materials support a broad range of disciplines and measurements including amount of substance, dimensional metrology, electricity and magnetism, mass and related quantities, time and frequency, among others [23]. Biomanufacturing is an expanding priority area for NIST as demonstrated by numerous recent publications on emerging scientific technologies including high resolution protein structure measurements [4–7], mass spectral libraries [24], rheology and behavior of high concentration protein solutions [25], protein particulates [26, 27], and cell therapies [28]. A key component of NIST’s Biomanufacturing Initiative is the development of industry-focused RMs designed to enable more accurate and confident characterization of key attributes linked to product safety and efficacy. NIST RMs are intended to play a unique role in the pre-competitive space where comprehensive characterization data can be freely disseminated and discussed for industry-wide collaboration, comparison, and open innovation.

The role of NIST RMs to support the biopharmaceutical sector was initially explored in 2009 at a US House of Representative Committee on Science and Technology Subcommittee Hearing on the “Potential Need for Measurement Standards to Facilitate the Research and Development of Biologic Drugs”. The seed was planted for a concerted effort toward the development of appropriate standards and has continued to grow through numerous workshops, seminars, and round-table discussions. Research at NIST was initiated to evaluate various measurement needs including potential glycoanalytical standards in support of the biomanufacturing industry; which ultimately concluded that a monoclonal antibody standard would best support the array of state-of-the-art technologies [29]. A developmental lot of the NISTmAb, designated as primary sample 8670 (PS 8670), has since served as the foundation for a cross-industry collaboration involving over 100 scientists evaluating the properties and suitability of the material [30–32]. Analytical and biophysical characterization spanning state-of-the art and emerging technologies was used to simultaneously establish a historical baseline dataset as well as confirm the NISTmAb’s industrial relevance [30–32]. In parallel, researchers at NIST made judicious use of the PS 8670 material to invent and advance biopharmaceutical-focused applications using unique NIST resources such as of NMR spectral fingerprinting and related higher-order structural technologies [4–6]. A resounding interest in a common, open innovation material was reinforced by studies on PS 8670, and vialing was henceforth pursued to enable a longitudinally available, homogeneous, and stable RM for public release.

The NISTmAb RM 8671, which is the topic of this publication series, was produced in murine suspension culture using the same industry standard upstream and downstream processing as PS 8670, differing only in homogenization of multiple bulk containers and fill-finish to suit the necessary lifecycle plan as described in more detail below. RM 8671 is a product neutral, class-specific (as opposed to product-specific) embodiment of the characteristics of IgG1κ therapeutics. It is composed of two heavy chains and two light chains interconnected with the canonical disulfide bonds to result in an ≈150 kDa homodimer. The primary amino acid sequence is decorated with post-translational modifications common to the IgG1κ class including heavy chain glycosylation, partial processing of C-terminal lysine, and low abundance oxidation, deamidation, and glycation [10, 14, 33, 34]. The class-representative product quality attributes and pre-competitive nature of the NISTmAb make it well suited as an industry-wide test metric for state-of-the-art and emerging measurement technologies. The next sections summarize the lifecycle management and quality control plan for the NISTmAb RM 8671, intended to ensure long-term availability and maintain consistent product quality attributes. The plan integrates ICH guidance documents and biopharmaceutical industry best practices for in-house reference standards with the NIST RM quality management paradigm. Method qualification, reference value assignment and stability evaluation exercises using various orthogonal approaches to determine concentration, identity, size heterogeneity, and charge heterogeneity are described in subsequent manuscripts in this series.

### Biopharmaceutical industry lifecycle management

Elements of a successful lifecycle management plan are presented throughout ICH guidance documents, and discussed most heavily in what might be considered the more “programmatic” ICH documents covering Pharmaceutical Development (Q8), Risk Management (Q9), Quality System (Q10), and Drug Substance Development and Manufacture (Q11). The following is intended to provide context for the NISTmAb lifecycle management plan described in the next section; the reader is directed to the suite of ICH documents (http://www.ich.org/products/guidelines/quality/article/quality-guidelines.html) for thorough guidance.

The “lifecycle” of a biopharmaceutical product spans the entirety of activities from initial research and development, transfer to manufacturing, commercial manufacturing, and the eventual discontinuation of a product from the market [35]. The pharmaceutical development stage is an information-rich timeframe wherein manufacturing capabilities, formulation, and analytical method development are optimized in a manner intended to meet the requirements for regulatory approval and patient need. Optimization activities may result in processes that generate products with a range of product quality attributes. Attributes of a given biopharmaceutical are often ranked on a continuum of criticality based on known or suspected structure-function relationship and correlation with manufacturing process variations [36]. This information is effective for building a knowledge space wider than the ultimate target product profile which can be leveraged for defining operating space, risk management, post-approval submissions, process improvements, etc. The final goal is to achieve a highly controlled process that delivers a constant product with a quality target product profile including critical quality attributes (CQAs). The optimized process and product testing protocols are transferred as necessary to extend the development stage into a commercial manufacturing realm.

Commercial manufacturing requires appropriate control (e.g., raw materials and process), production to meet market demand, quality control and assurance, and distribution [35]. It is at this stage where manufacturing historical knowledge increases significantly and can fuel future science and risk-based decisions. Process changes such as raw material sourcing, manufacturing sites or processes, are inevitable. Systematic knowledge management throughout a product’s lifecycle provides an opportunity for continuous development and improvement [37, 38]. A concept paper was recently introduced to begin development of ICH Q12: Technical and Regulatory Considerations for Pharmaceutical Product Lifecycle Management which is intended to enhance post-approval lifecycle guidance [39]. The document is envisioned to provide a framework for change management to the lifecycle plan and may also facilitate assimilation of novel control strategies, analytical procedures, and process tools as they become available to the industry. At some stage, the product may reach the end of its lifecycle, as may be the case when improved products are developed. A smooth transition involves retention of the historical product knowledge and continued stability evaluation to allow phasing out with minimal impact.

**Fig. 1:**
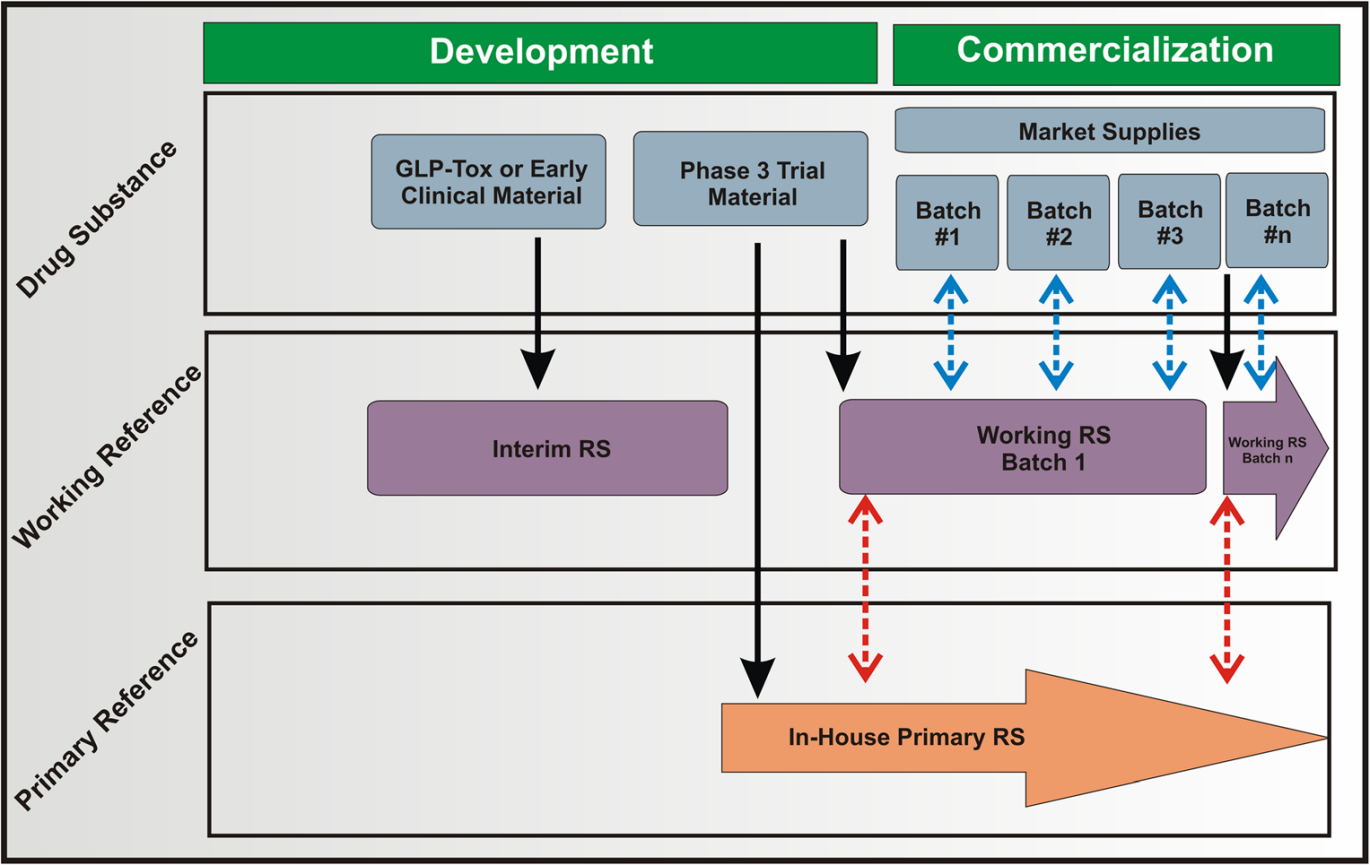
Representative lifecycle management plan employed by the biopharmaceutical industry that incorporates a two-tier reference standard. Solid arrows represent use of a material for a specific purpose. Dashed red arrow represents a working reference standard qualification exercise and dashed blue arrows represent lot release

Throughout the product lifecycle, the demand on the process is to produce a consistent product that meets the (phase appropriate) product quality target profile. An in-house reference standard, an appropriately characterized lot of the specific drug substance or drug product, is a logical comparator for helping to assure these criteria are met. This product-specific “gold standard” material run alongside new batches ensures system suitability of analytical testing results and continuity in product quality, stability, and comparability throughout the lifecycle [40]. Implementation of in-house reference standards early in the lifecycle allows for the most information-rich development stage. Replacement of in-house reference standards should be minimized. A representative phase appropriate two-tier implementation strategy is shown in Fig. 1 with this requirement in mind [13, 40].

A representative lot of early developmental material, often a Good Laboratory Practice (GLP) lot used for initial Phase 1 testing, is reserved as the interim in-house reference standard. This material should reflect the manufacturing process used in the early clinical trials and may require replacement should product attributes change due to process improvements and/or changes [40]. At or near phase 3 clinical trials, the process and product should be representative of that expected for the duration of the development phase as well as commercialization. At this point the two-tier aspect of the lifecycle plan may be enacted by reserving portions of a given lot as the in-house primary standard and the in-house working standard, a practice recommended in ICH Q6B [41]. Appropriate acceptance criteria established on the process/product-specific behavior enables direct comparison of analytical results from the in-house reference standard to commercial lots [42]. Primary and initial working standards are often reserved from the same lot of clinical material to ensure comparability. The working standard is then used as the routine control material for system suitability and lot release. A sufficient quantity of primary standard is reserved to allow qualification of future working standard lots as necessary due to exhaustion. This strategy minimizes longitudinal drift by maintaining a consistent primary standard for long term commercialization. Control data should be recorded from use of the working and primary standard materials to assess stability and/or monitor trends in the process or requalification of new working standards. Biosimilar manufacturers are held to the same expectations for an in-house reference standard program. Establishment of biosimilarity to originator marketed product is initially performed, yet the biosimilar manufacturer must utilize an in-house reference standard lifecycle management plan using lots manufactured with their specific process [43].

The notion of pooling production lots of material as an in-house reference standard has been explored [40]. Pooling is not necessary, but may be useful to ensure sufficiently large repository of in-house reference standard and to ensure that specific attributes are at an acceptable level (e.g., near the center of experience/acceptance range). All lots for pooling, however, should meet product acceptance criteria for attributes and stability. When available and fit-for-purpose, the in-house reference standards and in-house potency assay should be calibrated against a pharmacopeia or international material. This scenario is less common because an official product-specific RM is most often not available for recombinant biopharmaceutical products [43].

Analytical method lifecycle management shares many common features including evaluation and documentation of the capabilities and performance of a given a method throughout its historical use [13, 44]. Analytical knowledge management includes both method qualification, suitability assessment of a method, as well as method validation as outlined in ICH Q2(R1). Method accuracy, precision, specificity, detection limits, linearity, and range may be evaluated during validation depending on the method’s intended use [45]. Validated methods can then be used with an appropriate number of representative production lots to establish acceptance criteria for a given product [41]. Product specifications include analytical procedures and appropriate numerical limits, ranges or other criteria which a product must meet to be deemed acceptable for commercial release [41]. Not all analytical assays critical to a quality lifecycle management plan are fully validated and become part of release specifications. Rather, specifications are chosen to focus on the attributes found to be most useful in ensuring safety, purity, and efficacy rather than to establish full characterization data for each lot [41].

Characterization methods not chosen for lot release are still integral to the lifecycle management plan and may be used on a periodic basis to ensure integrity of in-house reference standards. Detailed characterization methods may be qualified as fit-for-purpose in evaluating properties such as higher order structure (e.g., NMR, HDX, CD, DSC) and specific post-translational modifications (e.g., mass spectrometry), among others [13]. Qualified characterization methods are often used for elucidation of structure to demonstrate a high level of product knowledge, and verify that robust QC methods are fit for their intended purpose [46]. Qualified characterization methods are required for qualification of future lots of in-house working standards and are critical to successful demonstration of biosimilarity. Trending characterization data associated with sequential production lots is also critical to post-approval comparability exercises that may be necessary to justify that process changes have not adversely impacted the product.

Akin to continuous process improvement, novel analytical and biophysical technologies become available throughout a product’s lifecycle. It is almost a certainty that a successful drug product will at some time require a method bridging study to implement an advanced technology or account for a method change [44]. Tracking and trending of in-house reference standards provide a means by which novel analytical technology can be assessed and implemented within a single company. Prior to the NISTmAb, however, there was no relevant Reference Material free of IP and/or regulatory concerns that could be used to openly develop and evaluate novel technology and serve as an external system suitability control. A comprehensive lifecycle management incorporating elements of a two-tiered reference standard and analytical quality monitoring program were implemented for the NISTmAb to ensure longitudinal availability of such a material.

### Lifecycle management plan for NISTmAb RM 8671

**Fig. 2:**
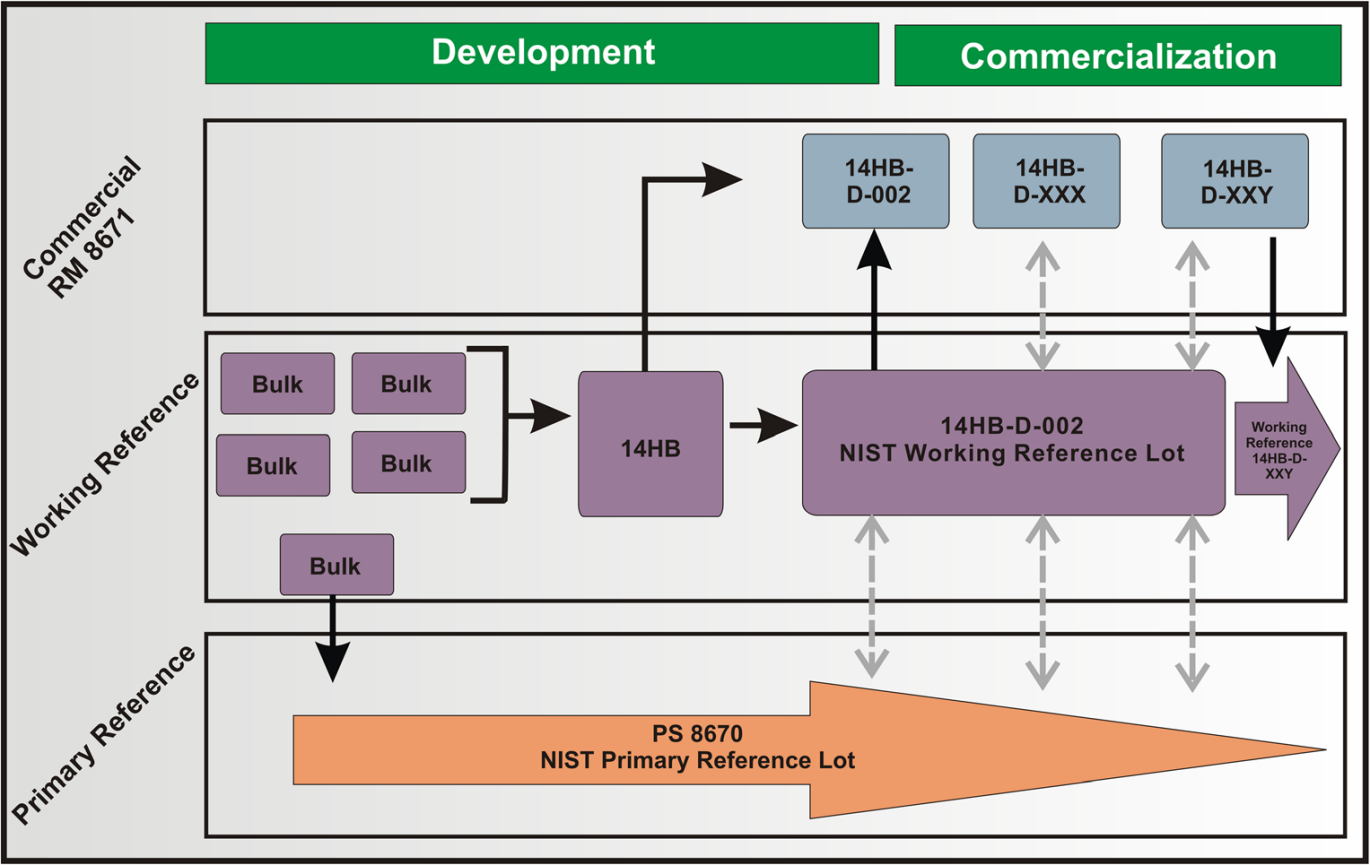
Lifecycle management plan for NISTmAb RM 8671. Solid arrows represent use of material to mix, vial, dilute, etc. Dashed gray arrow represents a value assignment exercise

The lifecycle management plan for the NISTmAb RM 8671 was modeled after ICH Guidance Documents and the two-tiered reference plan commonly used in the biopharmaceutical industry. The lifecycle plan contains the same elements outlined in ICH Q10: development, transfer, commercialization, and (if/when necessary) discontinuation. As will become evident, however, material intended as a public RM requires a few unique operations to ensure suitability for its purpose. For example, sufficient quantity of material was pooled prior to commercialization to avoid continued bio-production, such that “commercial manufacturing” will be solely fill finish. The structure of the lifecycle plan is intended to assure long-term availability of the material as well as consistent content and quality as shown in Fig. 2, with each element described in more detail below.

The *Development Stage* relies largely on production of a high quality, stable product. Typical production of biotherapeutic proteins is done in a batch process, wherein a series of unit operations are performed in tandem to produce a single batch, and repeated as necessary to meet market demand. The NISTmAb lifecycle management plan is unique in that the entirety of material (multiple “bulk” containers in Fig. 2) were prepared at the onset of the project rather than planning to produce additional batches moving forward. The bulk material was received as an ≈100 mg/mL frozen liquid and formulated in 12.5 mmol L-His, 12.5 mmol L-His HCl at pH 6.0 (referred to hereafter as formulation buffer). No additional excipients were present in the bulk material. At this stage an interim material was prepared at NIST from a single bulk container by diluting 10-fold with formulation buffer and vialing in screw-top cryovials at 1 mL per unit (approximately 10 mg/mL). This material was initially intended to serve as the interim primary reference sample as well as the preliminary test material for suitability evaluations. This material was utilized during the development stage for a variety of research purposes, and was initially identified as “candidate RM 8670 (lot 31fb)” [13]. This particular lot ultimately was reserved as the final in-house primary reference standard rather than being released as an RM. It will therefore be referred to from this point on as Primary Sample 8670 (PS 8670).

Preliminary characterization of the NISTmAb PS 8670 was performed by key stakeholders in an inter-laboratory collaboration. Participants included biopharmaceutical industry scientists, regulatory experts, and academic researchers with expertise in the entire spectrum of analytical and biophysical methods required for elucidation of structure. In many cases, multiple participants were recruited for a given attribute area to identify best practices and obtain harmonized results. The PS 8670 inter-laboratory crowdsourcing characterization is summarized in the ACS book series “State-of-the-art and emerging technologies for therapeutic monoclonal antibody characterization” [30–32]. This cross-industry study served to evaluate the material’s suitability as an RM as well as build a significant analytical and biophysical knowledgebase which is a unique advantage of this material. Additional analytical method development and qualification activities performed at NIST to establish PS 8670 are summarized below and detailed in additional papers in this series [14, 16, 17]. PS 8670 is therefore the lot for which NIST and its collaborators have the most historical product knowledge. It was for this reason PS 8670 was selected as the primary reference to which all future lots will be compared; predominantly as a system suitability material during value assignment of new fill-finish lots of material.

The successful inter-laboratory project confirmed the utility of the NISTmAb as a class-specific mAb and emphasized the importance of consistent product attributes. Despite rigorous process control, minor batch to batch heterogeneity may exist resulting in subtle changes in the product attributes, albeit within the normal experience of product related substances. The NISTmAb, however, is intended to be a physicochemical RM with consistent property values (e.g., concentration) and product quality attributes (size, charge, etc.). Therefore, a series of bulk containers were homogenized (pooled) in an effort to obtain a long-term supply of homogeneous material. The homogenization was performed to mix any batch to batch heterogeneity equally across the pooled sample, providing a large stock of consistent material (details of homogenization and fill-finish are included in the Electronic Supplementary Material (ESM)). Briefly, multiple bulk substance containers (≈100 mg/mL) were first homogenized to form the 14HB batch. Aliquots of 1 L were made from the homogenized bulk, designated as individual lots and frozen at −80 °C. Three lots (14HB-001, 14HB-002, and 14HB-003) were individually diluted 10 fold in formulation buffer and 800 μL aliquots placed into internally threaded screw top vials to be released as RM (lots 14HB-D-001, 14HB-D-002, and 14HB-D-003). Vials were placed in racks of 96 units each for storage at −80 °C. Sample processing was completed in a sterile environment using pre-sterilized single-use equipment and/or in a class 100,000 cleanroom environment. Three lots of material were initially vialed to both provide material for initial release as well as to give a measure of the inter-lot homogeneity that could be expected over time.

The *Commercialization* stage involves value assignment of commercial lots (to be sold on a cost recovery basis) as well as a continuous control strategy to ensure long term quality and availability. In the analogous stage of NISTmAb development, lot 14HB-D-002 was released as the first lot of official NISTmAb RM 8671. A portion of 14HB-D-002 was also set aside as the “working standard” and stored at −80 °C. The working standard is being used internally for routine evaluation of system performance, inter-laboratory comparisons, and research activities that operate at or below 10 mg/mL. PS 8670 is used as the system suitability standard when performing long term stability studies. The PS 8670 will also serve as the internal comparator during value assignment (described in more detail below) of new lots of material. It should be noted that the need to value assign a new lot of RM 8671 is expected to be infrequent in comparison to lot release demands of a marketed pharmaceutical product. We therefore expect that both the working standard and PS 8670 will be incorporated into future value assignments as system suitability controls. Combined, the lifecycle management plan set forth in Fig. 2 represents a two-tiered reference standard plan designed to ensure consistent attributes and the long term quality of RM 8671 for the foreseeable future.

During a typical biopharmaceutical lifecycle, a product may eventually be phased out of the market due to novel products that decrease patient need. RMs are seldom phased out unless there is no longer an industry need for such a material. This scenario is conceptually possible for the NISTmAb when biomanufacturing and product characterization have advanced to a stage where physicochemical and biological characterization comprehensively predicts clinical safety and efficacy. In this optimistic scenario, however, the NISTmAb may be even more useful as an external system suitability control in analytical evaluations. Should the time ever come that product discontinuation be considered, it will be evaluated in the same manner as the NISTmAb was created, in collaboration with a broad industry consortium to ensure no significant impact on the biomanufacturing industry.

### Quality plan for NISTmAb RM 8671

The NIST Quality Management (QM) system contains quality specific policies and procedures for acquisition of materials and supporting services including technical procedures and technical records [23]. The NISTmAb RM 8671 was issued under the QM system, thus its quality plan adheres to policies set forth for handling and storage, value and uncertainty budget assignment, as well as establishing homogeneity and stability [22, 23]. The following series of publications describes the procedures used to establish NISTmAb RM 8671 in accord with this QM system while incorporating concepts from ICH guidance documents for analytical method qualification and quality control [14–17]. The material was designated as fit for purpose, assigned reference and informational values, and evaluated for homogeneity and stability to allow broader utility of the material to the stakeholders. A brief description of the global quality plan is described here with pertinent definitions, followed by four publications describing the analytical methods, homogeneity assessment, stability assessment, and value assignment in detail [14–17].

Before going forward it is important to describe several NIST-specific definitions which are pertinent to the development of the NISTmAb. A NIST **Certified Value** represents the highest metrological designation wherein all known or suspected sources of bias have been fully investigated or accounted for and there is a high confidence in the reported value’s accuracy [47]. A NIST **Reference Value** is a noncertified value that is the best estimate of the true value based on available data; however, the value does not meet the NIST criteria for certification and is provided with associated uncertainty that may reflect only measurement precision, may not include all sources of uncertainty, or may reflect a lack of sufficient statistical agreement among multiple analytical methods [47]. **Informational Values** are reported in cases where uncertainty cannot be adequately assessed and/or only a limited number of analysis were performed, yet the experimental results may be highly valuable to the RM user. Information values cannot be used to establish metrological traceability [47]. A **Nominal Property** is one which is qualitative, for example the primary amino acid sequence is currently classified as such [48]. The NISTmAb was developed as described herein to meet the ISO definition of a Reference Material. RM 8671 is accompanied by a lot-specific report of investigation that demonstrates such, and includes reference values, informational values, and description of nominal properties [49]. Certification as a Certified Reference Material is a future endeavor that may be undertaken based on stakeholder feedback.

The NISTmAb RM 8671 report of investigation includes a reference value for mass concentration, informational values for hydrodynamic diameter and particle content, and confirmation of the dominant primary amino acid sequence [49]. A series of “physicochemical reference values” are also reported that were determined using qualified size exclusion chromatography (SEC), capillary electrophoresis – sodium dodecyl sulfate (CE-SDS), and capillary zone electrophoresis methods (CZE). While these reported values represent best estimates based on state-of-the-art measurements, they fall under the category of a reference value in that all sources of potential uncertainty have not been fully evaluated. They should not be taken as absolute values, but rather as method-specific reference values intended as a baseline for comparison of results from orthogonal, yet related assays that may be performed in a given user’s laboratory.

#### Reference value: Mass concentration value assignment

UV-visible spectrophotometry is commonly used to measure the spectral transmission of light at 280 nm. The negative base_10_ logarithm of spectral transmittance, decadic attenuance (D), includes the potential effects of scattering and luminescence upon the radiant power exiting the sample [50]. Concentrations of the NISTmAb RM 8671 are reported based on the decadic attenuance at 280 nm (D280). Measurements were performed on the NIST Transfer Spectrophotometer, which is traceable to the decadic logarithm of the derived SI unit of regular spectral transmittance through the control standard SRM 2031. Details on the experimental procedure and results for this analysis are contained in publication 5 of this series [15].

#### Physicochemical reference values and qualification plan

To ensure quality of the product and long term stability, a series of quality control assays must also be in place for monitoring quality attributes. Many of the methods described in the ACS book series were platform methods performed by the individual companies, and may be further optimized for the specific NISTmAb product and/or qualified for their intended use [33, 51]. The methodology employed in the ACS book series were therefore used as a historical baseline for further NISTmAb-specific optimization and qualification as set forth in publications 2 to 4 of this series [14, 16, 17]. Initial assays chosen for qualification were stability/quality-indicating assays, and additional assays are slated for incorporation into the NISTmAb quality management plan in the future. NISTmAb *qualification* exercises were designed to model ICH Q2 (R1), a documentary standard that sets forth considerations for the design and implementation of a related process, method *validation,* to demonstrate an analytical assay is suitable for its intended use [45]. Method qualification can be thought of as the analytical lifecycle stage that comes before validation. Elements of method qualification are not as well defined or harmonized as method validation, but have been described as the activities from optimization through readiness evaluation for a formal validation exercise [44]. Qualification may include, but is not necessarily limited to, method refinement and optimization, evaluation of stability/purity-indicating properties, and the collection of pre-validation data useful for setting expected acceptance criteria for formal validation exercises [44].

The general strategy taken for the NISTmAb was to first optimize and qualify the methods for PS 8670 to set method performance criteria as well as control limits to be used in system suitability evaluation during value assignment of individual RM 8671 lots. Method qualifications exercises are described in the following series of papers for stability/quality-indicating assays focusing on size heterogeneity profile (reduced and non-reduced CE-SDS and non-reduced SEC) and charge heterogeneity profile (CZE and capillary isoelectric focusing [CIEF]). Qualification involved optimization followed by determination of method figures of merit including linearity and range, limit of detection and limit of quantification (LOD and LOQ), specificity, accuracy, repeatability and intermediate precision. An overall strategy description is below, with a comprehensive description of method-specific details in publications 2 to 3 of this series [16, 17].

Methods were optimized using various responses as indicators of method performance including retention reproducibility, peak efficiency, percent area, and minimum sample preparation artifacts. For each qualified assay, an appropriate instrument qualification (IQ) marker (e.g., representative retention/migration time marker) was also evaluated throughout optimization and qualification. The IQ markers were selected to be free from sample preparation artifacts and primarily test the analytical instrumentation. The optimized methods were evaluated with one or more challenge samples (e.g., forced degradation material) to ensure the method can identify process- or product-related impurities and/or degradation products as necessary to be stability, purity, or identity indicating.

The linearity of an assay refers to its ability to produce results that are directly proportional to total amount of analyte in the sample [45]. Linearity was assessed for the main peak as well as any additional peak groups identified as product related substances. Sample levels were prepared at a range of at least 30% to 170% of the target loading concentration. Samples were prepared by dilution in most cases, however if neat sample was the target concentration, then bulk substance and/or injection quantity were options for preparation of samples. Method sample preparation was performed on each level in triplicate and injected in a randomized order along with appropriately placed sample matrix blank and IQ standard injections. Peak areas (or corrected peak areas for CE assays) were plotted individually versus nominal concentration to assess method linearity.

The LOD is the smallest amount of analyte that can be detected, but not necessarily quantified, and is commonly accepted as 3 times the noise level observed in an assay. Similarly, the LOQ is the lowest amount of analyte that can be reliably quantified with suitable precision, often defined as 10 times the noise level observed in the experiment. ICH Q2R1 provides several approaches that are acceptable for determination of LOD and LOQ, including the signal-to-noise ratio (SNR) approach used herein [45]. The LOD and LOQ were determined using a minor variant peak that could be reliably integrated but which had a low relative abundance with respect to other peaks. Optimally the SNR for the selected minor variant was within the range of 3 to 20 to most appropriately represent the expected performance of the integration parameters for a low concentration impurity. This peak was evaluated at the target loading concentration (C_target_) or a lower concentration within the linear range (C_inj_) such that the SNR was in the range of 3 to 20. The injection level chosen for LOD and LOQ determinations was required to be within the linear range for all peak groups identified in the assay at the target loading concentration. The LOD and LOQ were calculated using the SNR and percent relative abundance (RA) of the minor variant species as described in the ESM.

Specificity reflects the capability of a method to distinguish the measurand from impurities, degradants, and/or matrix interferences [45]. In the case of a purity assay, each of these species should also be quantifiable. NISTmAb assay specificity evaluation incorporated results from the forced degradation material injections as representative of potential impurities or degradants. Such an analysis serves as a specificity test because individual lots of RM 8671 will differ only in length of storage at −80 °C and date of vialing, therefore if any additional impurities are present in an individual lot they are expected to be product-related in nature. Specificity regarding matrix interference, injection carryover, and percent recovery was evaluated based on injections series of matrix blanks and one or more loading concentrations (e.g., blank-sample-blank).

Accuracy is the expression of the closeness of agreement between a measured value and the true (or conventionally accepted true) value [48, 52]. The conventional approach for demonstrating accuracy in purity measurements has relied on the use of small molecule primary calibrators in a mass balance approach [53]. Metrological traceability can be achieved for peptides and proteins via peptide impurity-corrected amino acid analysis [54–56]. In the case of qualified physicochemical assays describe herein, however, the intent is to provide method-specific reference quantity values in the form of relative charge or size purity values. The resultant reference values and associated uncertainties are intended as a basis for comparison with values of the same kind (as may be determined by a novel method in a stakeholder laboratory) for physicochemical attributes of the NISTmAb. Accuracy was therefore inferred from analytical method figures of merit (precision, linearity, and specificity) and comparison of orthogonal analytical procedures, per ICH Q2R1 [45].

The precision, or the closeness of agreement between replicate measurements, of each analytical method was evaluated by determination of repeatability and limited intermediate precision. Repeatability refers to replicate measurements using the same procedure, operators, and operating system over a short period of time [48, 52]. Repeatability was established via triplicate analysis of multiple analyte levels on one day during the linearity study. Intermediate precision is similar to repeatability; however, it incorporates other conditions that may experience change during a method’s lifecycle implementation [48, 52]. At a minimum the study design included factors such as multiple columns/capillaries, IQ standard lots, and gel/buffer lots over multiple days. Equipment and analyst were not varied (as would be required for full validation according to ICH Q2R1 [45]) due to resource limitations. The complete description of protocols and expected acceptance criteria detailed in the current publication series, however, provides an opportunity for such validation exercises after the RM is released; and may represent a platform for reproducibility testing (inter-laboratory studies) performed in conjunction with stakeholder labs should industry feedback deem them appropriate. A general description of the statistical treatment of PS 8670 qualification data for the purpose of setting method performance criteria is included in the ESM. Method performance criteria for each method are reported in the respective publication in this series, and were then used for system suitability evaluations during RM 8671 value assignment as described in the final publication of this series.

Method qualification/validation rigor is necessary in the regulatory environment to ensure consistent product quality. Although not intended to be a regulatory exercise or guidance as to how method qualification should be performed, the NISTmAb method qualification endeavors were performed in alignment with ICH Q2(R1) principles to operate under industry-relevant controls and simulate industrial challenges. The detailed analytical protocols and method performance criteria obtained during PS 8670 qualification are published in the following series [16, 17]. The physicochemical evaluation methods were then applied to three lots of NISTmAb RM 8671 to evaluate homogeneity, stability and assign lot-specific reference values as reported on the Report of Investigation available with the material and described in detail in publication 5 of this series [15, 49]. This analysis was performed while bracketing with the IQ standard specific to each method and PS 8670 to ensure system suitability; effectively qualifying each RM 8671 lot as fit-for-purpose.

#### Informational value assignment

As discussed in the Industry Lifecycle Management section, a cadre of characterization assays exists that are critical for evaluating the complexity of protein therapeutics. Comprehensive evaluation of size heterogeneity is one such attribute that requires multiple orthogonal techniques. The qualified methods described above are commonly applied as quality control assays, however, orthogonal assays may be used at various lifecycle stages and/or are necessary to cover the entire spectrum of potential size variants. Dynamic light scattering (DLS) is capable of sizing particles in the range of 1 nm to 1 μm in size and is commonly employed during high throughput developabilty studies. Flow imaging (FI) can be used for the analysis of subvisible particles (2 μm to 100 μm in size) in suspension. Considering the potential safety implications of aggregates and higher-order protein particulates [26, 27], the hydrodynamic diameter measured using DLS and subvisible particle concentration using FI results are included in the form of informational property values.

The approach taken for informational value assignment utilized many of the same measurement principles as described for the qualified physicochemical assays; Instrument system suitability criteria were established using an appropriate IQ standard, methods were optimized for PS 8670, and repeatability/intermediate precision assessment was performed [17]. The methods were then applied in the final publication to three lots of NISTmAb RM 8671 to evaluate homogeneity, stability and assign lot-specific informational values [15]. This analysis was performed while bracketing with the IQ standard specific to each method and PS 8670 to ensure system suitability; effectively qualifying each RM 8671 lot as fit-for-purpose.

#### Identity

Biopharmaceuticals are, at a basal level, a manifestation of their constituent primary amino acid sequence. The identity is therefore traditionally confirmed through elucidation of the primary structure. These relatively simplistic terms represent a complexity of astonishing proportion, illustrated by a theoretical estimation of over 10^8^ factorial combinations when considering only a subset of potential post-translational modifications [3]. This diversity is fortunately curbed by inherent biological control mechanisms and advanced biomanufacturing technologies; nevertheless it remains challenging to evaluate. The primary structure of PS 8670 was initially confirmed as a crowdsourced characterization effort as described in the ACS book series [33, 34]. Contributors utilized high resolution mass spectrometry of the intact protein and its subunits and ultrahigh-performance liquid chromatography with ultraviolet and high-resolution tandem mass spectrometry detection (LC-MS/MS peptide mapping). The identity of the NISTmAb was confirmed to be a class-representative IgG1κ, comprised of amino acid sequence, post translational modifications, and low abundance sequence variants typical of marketed IgG1 products.

LC-MS/MS remains the current state-of-the-art for primary sequence verification due to its sensitivity and selectivity, and hence selected for the NISTmAb control assay. Continued advancements in column chemistries, reagent quality, and instrument performance have advanced its robustness such that multiple attributes can be simultaneously monitored with high confidence [9]. The successful implementation of this information rich methodology relies heavily on a highly optimized and controlled enzymatic digestion protocol. In developing the optimized LC-MS/MS NISTmAb identity assay, each stage of trypsin digestion was critically evaluated for PS 8670 in an effort to minimize digest-induced artifacts while maintaining high cleavage efficiency (fourth publication of this series [14]). LC-MS/MS peptide mapping analysis of the PS 8670 digest provided a reference map to which future lots of RM 8671 could be compared. Sequence confirmation of RM 8671 included conformance to the reference peptide map with MS/MS peptide identifications, retention times, and relative peak abundances. This analysis was performed while bracketing with PS 8670 to ensure system suitability; effectively qualifying each RM 8671 lot as fit-for-purpose.

## Summary

The development and marketing of mAb and mAb-derived therapeutics will undoubtedly continue to grow, necessitating a parallel growth in available standards to support them. The spectrum of available documentary standards has surpassed that of physical standards, a dynamic that is beginning to change. Physical standards may at times have overlapping, yet cooperative applications in the biopharmaceutical setting, and the potential applications are still in their infancy. Initial uptake by the biopharmaceutical industry and feedback on performance (for both intended and off-label uses) are critical to evolution of available public standards and informing the next generation of materials. Common to all standards, both public and private, is the need for a comprehensive lifecycle management plan that ensures longitudinal quality and availability. This was accomplished for the NISTmAb via qualified assays to ensure stability, a market supply lineage for highly reproducible lots, and a two-tiered internal reference program for material qualification/comparison of released RM lots. The overview of the NISTmAb lifecycle plan described above will be further detailed in the following publications of this series, specifically focusing on qualification of control assays, confirmation of identity, and assignment of reference values for released lots; a data package that will inevitably be updated with additional technologies as its lifecycle continues.

The NISTmAb is an advance to the public standards paradigm, intended to serve as a model upon which a pre-competitive knowledgebase can be constructed. The NISTmAb is unique in that it is class-specific (as opposed to product-specific), has an openly available extensive characterization package, is released with numerous assigned reference values, and is voluntary in nature enabling suitability for a broad scope of applications. Potential utility includes assessing method variability in comparative analytical studies, serving as an external system suitability control, and facilitating a common framework for class-specific (e.g., platform) analytical assays. The NISTmAb is not intended to replace in-house product-specific reference standards; however, it may supplement aspects of method development, qualification and validation. The NISTmAb is also an industry-relevant sample for development and implementation of innovative technologies. It may serve as a representative test case, for example, to elucidate complex dataset integration frameworks and establishing their impact on comparability/biosimilarity assessment, de-risking of innovative technologies for lifecycle-appropriate implementation and regulatory assimilation, and as a harmonization tool for the continuously evolving best practices of the pharmaceutical industry. It is expected that introduction of this novel standard will lead to an increased awareness of the utility of open innovation standards and the eventual development of RMs for emerging complex biotherapeutic modalities, process developments tools, etc. where standards and pre-competitive collaboration show promise for transformative developments.

## Electronic supplementary material

ESM 1 (PDF 610 kb)

## References

[1] Elgundi Z, Reslan M, Cruz E, Sifniotis V, Kayser V. The state-of-play and future of antibody therapeutics. Adv Drug Deliv Rev. 2016; 10.1016/j.addr.2016.11.004. 10.1016/j.addr.2016.11.004 27916504

[2] Ecker DM, Jones SD, Levine HL (2015). The therapeutic monoclonal antibody market. MAbs.

[3] Kozlowski S, Swann P (2006). Current and future issues in the manufacturing and development of monoclonal antibodies. Adv Drug Deliv Rev.

[4] Arbogast LW, Brinson RG, Formolo T, Hoopes JT, Marino JP (2016). 2D (1)H(N), (15)N correlated NMR methods at natural abundance for obtaining structural maps and statistical comparability of monoclonal antibodies. Pharm Res.

[5] Arbogast LW, Brinson RG, Marino JP (2015). Mapping monoclonal antibody structure by 2D 13C NMR at natural abundance. Anal Chem.

[6] Arbogast LW, Brinson RG, Marino JP (2016). Application of natural isotopic abundance (1)H-(1)(3)C- and (1)H-(1)(5)N-correlated two-dimensional NMR for evaluation of the structure of protein therapeutics. Methods Enzymol.

[7] Marino JP, Brinson RG, Hudgens JW, Ladner JE, Gallagher DT, Gallagher ES et al. Emerging Technologies To Assess the Higher Order Structure of Monoclonal Antibodies. State-of-the-Art and Emerging Technologies for Therapeutic Monoclonal Antibody Characterization Volume 3. Defining the Next Generation of Analytical and Biophysical Techniques. ACS Symposium Series, vol 1202: American Chemical Society; 2015. p. 17–43.

[8] Campuzano IDG, Larriba C, Bagal D, Schnier PD. Ion Mobility and Mass Spectrometry Measurements of the Humanized IgGk NIST Monoclonal Antibody. State-of-the-Art and Emerging Technologies for Therapeutic Monoclonal Antibody Characterization Volume 3. Defining the Next Generation of Analytical and Biophysical Techniques. ACS Symposium Series, vol 1202: American Chemical Society; 2015. p. 75–112.

[9] Rogers RS, Nightlinger NS, Livingston B, Campbell P, Bailey R, Balland A (2015). Development of a quantitative mass spectrometry multi-attribute method for characterization, quality control testing and disposition of biologics. MAbs.

[10] Prien JM, Stöckmann H, Albrecht S, Martin SM, Varatta M, Furtado M et al. Orthogonal Technologies for NISTmAb N-Glycan Structure Elucidation and Quantitation. State-of-the-Art and Emerging Technologies for Therapeutic Monoclonal Antibody Characterization Volume 2. Biopharmaceutical Characterization: The NISTmAb Case Study. ACS Symposium Series, vol 1201: American Chemical Society; 2015. p. 185–235.

[11] Remmele RL, Bee JS, Phillips JJ, Mo WD, Higazi DR, Zhang J et al. Characterization of Monoclonal Antibody Aggregates and Emerging Technologies. State-of-the-Art and Emerging Technologies for Therapeutic Monoclonal Antibody Characterization Volume 3. Defining the Next Generation of Analytical and Biophysical Techniques. ACS Symposium Series, vol 1202: American Chemical Society; 2015. p. 113–58.

[12] FDA US (2015). Guidance for industry: scientific considerations in demonstrating biosimilarity to a reference product.

[13] Schiel JE, Mire-Sluis A, Davis DL, Schiel JE, Davis DL, Borisov OB (2014). Monoclonal antibody therapeutics: the need for biopharmaceutical reference materials. State-of-the-art and emerging Technologies for Therapeutic Monoclonal Antibody Characterization Volume 1. Monoclonal antibody therapeutics: structure, function, and regulatory space. ACS symposium series, vol 1176: American Chemical Society.

[14] Mouchahoir T, Schiel JE. Development of an LC-MS/MS peptide mapping protocol for the NISTmAb. Anal Bioanal Chem. 2018; 10.1007/s00216-018-0848-6. 10.1007/s00216-018-0848-6 PMC5830484 29411091

[15] Schiel JE, Turner A, Mouchahoir T, Yandrofski K, Telikepalli S, King J, DeRose P, Ripple D, Phinney K. The NISTmAb Reference Material 8671 value assignment, homogeneity, and stability. Anal Bioanal Chem. 2018; 10.1007/s00216-017-0800-1. 10.1007/s00216-017-0800-1 PMC5830482 29411089

[16] Turner A, Schiel JE. Qualification of NISTmAb charge heterogeneity control assays. Anal Bioanal Chem. 2018; 10.1007/s00216-017-0816-6. 10.1007/s00216-017-0816-6 PMC5830499 29423598

[17] Turner A, Yandrofski K, Telikepalli S, King J, Heckert A, Filliben J, Ripple D, Schiel J. Development of orthogonal NISTmAb size heterogeneity control methods. Anal Bioanal Chem. 2018; 10.1007/s00216-017-0819-3. 10.1007/s00216-017-0819-3 PMC5830496 29428991

[18] Cochrane RC (1966). Measures of progress: a history of the national bureau of standards.

[19] Joint Committee for Guides in Metrology. International vocabulatry of metrology (VIM) 200:2012. https://www.bipm.org/utils/common/documents/jcgm/JCGM_200_2012.pdf

[20] ISO.: ISO Guide 30, Reference materials-selected terms and definitions. Geneva: ISO. 2015.

[21] ISO.: ISO 17034, General requirements for the competence of reference material producers. Geneva: ISO. 2016.

[22] ISO.: ISO Guide 35. Reference materials - Guidance for characterization and assessment of homogeneity and stability. Geneva: ISO. 2017.

[23] Bruce S. The NIST quality system for measurement services: a look at its past decade and gaze towards its future. NCSLI International Workshop and Symposium. 2013. http://ws680.nist.gov/publication/get_pdf.cfm?pub_id=913859

[24] Dong Q, Yan X, Liang Y, Stein SE (2016). In-depth characterization and spectral library building of Glycopeptides in the tryptic digest of a monoclonal antibody using 1D and 2D LC-MS/MS. J Proteome Res.

[25] Dharmaraj VL, Godfrin PD, Liu Y, Hudson SD (2016). Rheology of clustering protein solutions. Biomicrofluidics.

[26] Ripple DC, Hu Z (2016). Correcting the relative bias of light obscuration and flow imaging particle counters. Pharm Res.

[27] Ripple DC, Narhi LO. Protein Particles (0.1 μm to 100 μm). State-of-the-Art and Emerging Technologies for Therapeutic Monoclonal Antibody Characterization Volume 2. Biopharmaceutical Characterization: The NISTmAb Case Study. ACS Symposium Series, vol 1201: American Chemical Society; 2015. p. 357–86.

[28] Simon CG, Lin-Gibson S, Elliott JT, Sarkar S, Plant AL (2016). Strategies for achieving measurement Assurance for Cell Therapy Products. Stem Cells Transl Med.

[29] Schiel JE, Au J, He HJ, Phinney KW (2012). LC-MS/MS biopharmaceutical glycoanalysis: identification of desirable reference material characteristics. Anal Bioanal Chem.

[30] Schiel JE, Davis DL, Borisov OB, editors.: State-of-the-Art and Emerging Technologies for Therapeutic Monoclonal Antibody Characterization Volume 1. Monoclonal Antibody Therapeutics: Structure, Function, and Regulatory Space. ACS Symposium Series, vol 1176: American Chemical Society; 2014. p. 165.

[31] Schiel JE, Davis DL, Borisov OB, editors.: State-of-the-art and emerging technologies for therapeutic monoclonal antibody characterization volume 3. Defining the next generation of analytical and biophysical techniques. ACS Symposium Series, vol 1202: American Chemical Society; 2015. p. 455.

[32] Schiel JE, Davis DL, Borisov OB, editors. State-of-the-art and emerging technologies for therapeutic monoclonal antibody characterization volume 2. biopharmaceutical characterization: The nistmab case study. ACS Symposium Series, vol 1201: American Chemical Society; 2015. p. 427.

[33] Formolo T, Ly M, Levy M, Kilpatrick L, Lute S, Phinney K et al. Determination of the NISTmAb Primary Structure. State-of-the-Art and Emerging Technologies for Therapeutic Monoclonal Antibody Characterization Volume 2. Biopharmaceutical Characterization: The NISTmAb Case Study. ACS Symposium Series, vol 1201: American Chemical Society; 2015. p. 1–62.

[34] Li W, Kerwin JL, Schiel J, Formolo T, Davis D, Mahan A et al. Structural Elucidation of Post-Translational Modifications in Monoclonal Antibodies. State-of-the-Art and Emerging Technologies for Therapeutic Monoclonal Antibody Characterization Volume 2. Biopharmaceutical Characterization: The NISTmAb Case Study. ACS Symposium Series, vol 1201: American Chemical Society; 2015. p. 119–83.

[35] ICH. Q10 Pharmacuetical Quality System. ICH Harmonised Tripartite Guidline 2008.

[36] Flynn GC, Nyberg GB. Using Quality by Design Principles in Setting a Control Strategy for Product Quality Attributes. State-of-the-Art and Emerging Technologies for Therapeutic Monoclonal Antibody Characterization Volume 1. Monoclonal Antibody Therapeutics: Structure, Function, and Regulatory Space. ACS Symposium Series, vol 1176: American Chemical Society; 2014. p. 117–50.

[37] International Counsil for Harmonisation.. Q11 Development and manufacture of drug substances. ICH Harmonised Tripartite Guidline. 2012. http://www.ich.org/fileadmin/Public_Web_Site/ICH_Products/Guidelines/Quality/Q11/Q11_Step_4.pdf.

[38] A lifecycle approach to knowledge excellence in the biopharmaceutical industry. Boca Raton: CRC Press; 2017.

[39] International Counsil for Harmonisation.. Q12 Final Concept Paper: Technical and regulatory considerations for pharmaceutical prodcut lifecycle management. ICH Harmonised Tripartite Guidline. 2015. http://www.ich.org/fileadmin/Public_Web_Site/ICH_Products/Guidelines/Quality/Q12/Q12_Final_Concept_Paper_July_2014.pdf.

[40] Mire-Sluis A, Ritter N, Cherney B, Schmalzing D, Blumel M (2014). Reference standards for therapeutic proteins part 1. Bioprocess International..

[41] International Counsil for Harmonisation. Q6B Specifications: Test procedures and acceptance criteria for biotechnological products. ICH Harmonised Tripartite Guidline. 1999. http://www.ich.org/fileadmin/Public_Web_Site/ICH_Products/Guidelines/Quality/Q6B/Step4/Q6B_Guideline.pdf.

[42] Esterman AL, Katiyar A, Krishnamurthy G (2016). Implementation of USP antibody standard for system suitability in capillary electrophoresis sodium dodecyl sulfate (CE-SDS) for release and stability methods. J Pharm Biomed Anal.

[43] Mire-Sluis A, Ritter N, Cherney B, Schmalzing D, Blumel M (2014). Reference standards for therapeutic proteins part 2. Bioprocess International.

[44] Douette P, Bolon P. Analytical method lifecycle: a roadmap for biopharmaceutical development. Biopharm International. 2013:46–53.

[45] International Counsil for Harmonisation. Q2(R1) Validation of analytical procedures: Text and methodology. ICH Harmonised Tripartite Guideline. 2005. http://www.ich.org/fileadmin/Public_Web_Site/ICH_Products/Guidelines/Quality/Q2_R1/Step4/Q2_R1__Guideline.pdf

[46] International Counsil for Harmonisation. M4Q(R1) The common technical document for the registration of pharmaceuticals for human use: Quality. ICH Harmonised Tripartite Guidline. 2002. http://www.ich.org/fileadmin/Public_Web_Site/ICH_Products/CTD/M4_R1_Quality/M4Q__R1_.pdf.

[47] May W, Parris R, Beck C II, Fassett J, Greenberg R, Guenther F, et al. Definitions of terms and modes used at NIST for value-assignment of reference materials. Special publication 260-136. Washington, DC: government printing. Office. 2000;

[48] 200:2012 J. International Vocabulary of Metrology-Basic and General Concepts and Associated Terms (VIM 3rd edition. Joint Committee for Guides in Metrology.

[49] Report of Investigation: Reference Material 8671. https://www-s.nist.gov/srmors/view_detail.cfm?srm=8671. Accessed December 2017.

[50] IUPAC Compendium of Chemcal terminology. 2 ed. 1997.

[51] Michels DA, Ip AY, Dillon TM, Brorson K, Lute S, Chavez B, Schiel JE, Davis DL, Borisov OB (2015). Separation methods and orthogonal techniques. State-of-the-art and emerging Technologies for Therapeutic Monoclonal Antibody Characterization Volume 2. Biopharmaceutical characterization: the NISTmAb case study. ACS symposium series, vol 1201: American Chemical Society.

[52] Joint Committee for Guides in Metrology. Evaluation of Measurement Data - Guide to the expression of uncertainty in measurement (GUM 1995 With minor additions). 200:2008. https://www.bipm.org/en/publications/guides/gum.html.

[53] Westwood S, Choteau T, Daireaux A, Josephs RD, Wielgosz RI (2013). Mass balance method for the SI value assignment of the purity of organic compounds. Anal Chem.

[54] Munoz A, Kral R, Schimmel H (2011). Quantification of protein calibrants by amino acid analysis using isotope dilution mass spectrometry. Anal Biochem.

[55] Stoppacher N, Josephs RD, Daireaux A, Choteau T, Westwood S, Wielgosz RI (2015). Accurate quantification of impurities in pure peptide material - angiotensin I: comparison of calibration requirements and method performance characteristics of liquid chromatography coupled to hybrid tandem mass spectrometry and linear ion trap high-resolution mass spectrometry. Rapid Commun Mass Spectrom.

[56] Stoppacher N, Josephs RD, Daireaux A, Choteau T, Westwood SW, Wielgosz RI (2013). Impurity identification and determination for the peptide hormone angiotensin I by liquid chromatography-high-resolution tandem mass spectrometry and the metrological impact on value assignments by amino acid analysis. Anal Bioanal Chem.

