# Supplementary material for: The NISTmAb Reference Material 8671 lifecycle management and quality plan
Source: Anal Bioanal Chem. 2018 Feb 12;410(8):2067–78. doi: 10.1007/s00216-017-0844-2 (PMC5830479; doi:10.1007/s00216-017-0844-2)
Supplement: Supplementary file 1 — (PDF 610 kb) [file 216_2017_844_MOESM1_ESM.pdf]

Analytical and Bioanalytical Chemistry

Electronic Supplementary Material

The NISTmAb Reference Material 8671 lifecycle management and quality plan

John E. Schiel, Abigail Turner

14HB Prepration. Bulk homogenization to prepare 14HB was performed by Akron Biosciences (Boca Raton, FL); the protocol utilized is briefly summarized here. The bulk containers were moved to ambient storage to begin thawing with a fan utilized to circulate ambient air in the room. The bulk was thawed at ambient temperature (recorded between 20° C and 22° C) for 2 hours and 45 minutes. The bulk containers were then approximately 70% thawed and were moved to 4° C storage. The thawing was completed by storing them at 4° C for approximately 19 hours and 30 minutes. The bulk containers were then sterilized with isopropyl alcohol and moved into the ISO class 7 cleanroom. Bulk material from each container was transferred to a sterile mixing Cellbag (GE Cellbag Model CB0100L10, 100 L capacity) in the cleanroom. The bulk containers were connected in series by a sterile manifold consisting of 5 lengths of silicon tubing to the sterile mixing Cellbag. The sterile mixing Cellbag was secured inside a GE Wave Bioreactor (Model 200 EH). The terminal end of the transfer manifold was connected to the inlet of the sterile mixing Cellbag. The peristaltic pump was started (175 mL/min) and bulk material was transferred from the Celsius-Pak into the mixing Cellbag. Transfer from the bulk to the Mixing Cellbag was at ambient temperature and completed in a total time of four hours.

Mixing and bottling were performed after all material had been transferred to the mixing Cellbag. The bulk material was homogenized by mixing on the Wave 200 for one hour at ambient temperature. The mixing was set at 4 rocks/min at an angle of 2°. At the completion of mixing, the outlet tube of the mixing Cellbag was threaded into the head of the peristaltic pump and the end of the outlet tube was suspended inside a laminar flow hood. The homogenized bulk was transferred from the mixing Cellbag into gamma sterilized polycarbonate biotainers (Nalgene, part number 3120-42). The peristaltic pump was set to transfer the homogeneous bulk

at a rate of 150 mL/min. As the biotainers were filled, they were removed from the laminar flow hood, labeled sequentially, and transferred to -80º C storage. Filling was completed in 2 hours and 30 minutes. Each of these bulk containers are part of 14-Homogenized Bulk (14HB), labeled as 14HB-XXX where XXX is the designated lot number in the sequence they were filled.

Dilution and Vialing of 14HB-D-001, 14HB-D-002, and 14HB-D-003. Each 14HB container can be individually thawed to make RM and/or SRM dilution lots as 14-HB-D-XXX where "D-XXX" is the dilution and lot number. Dilution and vialing of three of the 14HB lots was performed by Bioserve (San Diego, CA); the protocol utilized is briefly summarized here. Formulation buffer for dilution (12.5 mM L-His, 12.5 mmol L-His HCl, pH 6.0) was prepared using sterile water for injection (Baxter, PN 2B0307), L-histidine monohydrochloride (J.T. Baker, PN 2081-06), and L-histidine (J.T. Baker, PN 2080-05). In summary, a single container of 14HB was thawed in a 25° C water bath. The 14HB was diluted by mass using formulation buffer to an approximate final target concentration of 10 mg/mL in a carboy, resulting in 14HB-D-XXX. The carboy was gently mixed with stirring while the fill took place to ensure homogeneity of the fill across all vials of a given lot. A Thermo CapItAll capper/decapper (Nalge Nunc, Cat. No. 4111MAT) was used to decap the prelabeled vials (Thermo Matrix, PN 3741). A Thermo Multidrop Combi Dispenser (Molecular BioProduct, Cat. No 5840300 with standard tube dispensing cassette, Cat. No. 24072670) was utilized to automatically transfer 800 µL of 14HB-D-XXX to vials of a given rack. The filled rack was then re-capped in the CapItAll capper. Each rack was weight checked to ensure appropriate fill volume had been added to a given rack. Racks were flash frozen on dry ice and then transferred to -80° C storage until shipment. The dilution and vialing was individually performed on three of the 14HB lots to produce 14HB-D-001, 14HB-D-002, and 14HB-D-003. Three lots of material were vialed to

both provide material for initial release as well as to give a measure of the inter-lot homogeneity that could be expected over time.

Method for Limit of Detection (LOD) and Limit of Quantification (LOQ) Calculation. The LOD and LOQ were calculated using the SNR and percent relative abundance (RA) of the minor variant species according to Equations 1 and 2.

$$LOD(mg) = \frac{3}{SNR_{mv}} \times \frac{RA_{mv}(\%)}{100} \times C_{inj} \times V_{inj} \quad (1)$$

$$LOQ(mg) = \frac{10}{SNR_{mv}} \times \frac{RA_{mv}(\%)}{100} \times C_{inj} \times V_{inj} \quad (2)$$

where C_{inj} is concentration of total protein loaded in the experiment (mg/mL) and V_{inj} is the injection volume (mL). The mass-based LOD and LOQ were then converted to percent (%) relative abundance corresponding to the experiment run at the target concentration using equations 3 and 4.

$$LOD(\%) = \frac{LOD}{C_{target} \times V_{inj}} \times 100 \quad (3)$$

$$LOQ(\%) = \frac{LOQ}{C_{target} \times V_{inj}} \times 100 \quad (4)$$

In the case of the CE assays, $C_{inj} = C_{target}$ because a minor variant (e.g., NGH) was present at appropriate SNR for this type of determination. This may not be true for all analytes and all assay types (as will be seen for SEC of the NISTmAb). The two-step calculation method described allows for mass-based LOD and LOQ to be calculated at a C_{inj} smaller than the C_{target} (but still within the linear range) and later converted to a percent-based LOD/LOQ at the target loading concentration (C_{target}) of the optimized assay.

Statistical Treatment 8670 Qualification Data. The total variance of analysis conducted on PS 8670 has potential contributions from the filling process, repeatability of the analytical method, and intermediate precision of the analytical method (column, day, buffer lot, etc.). Each of the intermediate precision factors was held constant within a given day, and therefore can be considered as a day to day variability, identified at $u^2_{other,8670}$. The total variance of an analytical method performed on 8670 is therefore:

$$u^2_{c,8670} = u^2_{\mathrm{fill},8670} + u^2_{r,8670} + u^2_{other,8670} \tag{5}$$

Where $u_{\mathrm{c},8670}$ represents the intermediate precision of the assay reported as a combined standard uncertainty (at the level of one standard deviation). For all qualification exercises, 8670 was reserved from the same rack of material, in which case the fill variance can be considered negligible, which reduced equation 5 to equation 6.

$$u^2_{c,8670} = u^2_{r,8670} + u^2_{other,8670} \tag{6}$$

Because $u^2_{other,8670}$ cannot be measured directly, it must be estimated from repeatability measured on multiple days and columns (intermediate precision conditions). This was done using a one-way nested ANOVA to split the measured variances into "within group" (or repeatability, $u^2_{r,8670}$) and "between group" ($u^2_{other,8670}$) contributions to the total variance as given in (2). The ANOVA returned a "between group mean square" (BMS) that is equivalent to

$$E(BMS) = n \times u^2_{other,8670} + u^2_{r,8670} \tag{7}$$

where n is the number of observations in each repeatability measurement. Solving for $u^2_{other,8670}$ (which will be used in assigning uncertainty budget for RM 8671 as described in the final publication of this series) [1] and inserting into Equation 6 gives the total intermediate precision

of the method for 8670. Results from ANOVA analysis were used to set the method performance criteria for PS 8670 as $\pm 3u_{c,8670}$. A similar analysis was performed to set method performance criteria for IQ standards. Method performance criteria for each method are reported in the respective publication in this series, and were then used for system suitability evaluations during RM 8671 value assignment as described in the final publication of this series.

1. Schiel JE, Turner A, Mouchahoir T, Yandrofski K, Telikepalli S, King J et al. The NISTmAb Reference Material 8671 Value Assignment, Homogeneity, and Stability. Anal Bioanal Chem. 10.1007/s00216-017-0800-1.
